# Subclinical Hypothyroidism in Moderate-to-Severe Psoriasis: A Cross-Sectional Study of Prevalence and Clinical Implications

**DOI:** 10.3390/diseases13080237

**Published:** 2025-07-25

**Authors:** Ricardo Ruiz-Villaverde, Marta Cebolla-Verdugo, Carlos Llamas-Segura, Pedro José Ezomo-Gervilla, Jose Molina-Espinosa, Jose Carlos Ruiz-Carrascosa

**Affiliations:** 1 Department of Dermatology, Hospital Universitario San Cecilio, 18016 Granada, Spain; marta.cebolla.sspa@juntadeandalucia.es (M.C.-V.); carlos.llamas.sspa@juntadeandalucia.es (C.L.-S.); pedroj.ezomo.sspa@juntadeandalucia.es (P.J.E.-G.); jose.molina.espinosa.sspa@juntadeandalucia.es (J.M.-E.); jcarlos.ruiz.sspa@juntadeandalucia.es (J.C.R.-C.); 2 Instituto Biosanitario de Granada (ibs.GRANADA), 18014 Granada, Spain

**Keywords:** Psoriasis, hypothyroidism, Epidemiology

## Abstract

**Background**: Psoriasis is a chronic inflammatory skin disease linked to systemic comorbidities, including metabolic, cardiovascular, and autoimmune disorders. Thyroid dysfunction, particularly hypothyroidism, has been observed in patients with moderate-to-severe psoriasis, suggesting possible shared inflammatory pathways. **Objectives**: This study aims to explore the relationship between psoriasis and thyroid dysfunction in adults with moderate-to-severe psoriasis undergoing biologic therapy to determine whether psoriasis predisposes individuals to thyroid disorders and to identify demographic or clinical factors influencing this association. **Materials and Methods**: A cross-sectional study included adult patients with moderate-to-severe psoriasis receiving biologic therapy, recruited from the Psoriasis Unit at the Dermatology Department of Hospital Universitario San Cecilio in Granada, Spain, from 2017 to 2023. Patients with mild psoriasis or those treated with conventional systemic therapies were excluded. The data collected included demographics and clinical characteristics, such as age, sex, BMI (body mass index), and psoriasis severity (psoriasis severity was evaluated using the Psoriasis Area Severity Index (PASI), body surface area (BSA) involvement, Investigator’s Global Assessment (IGA), pruritus severity using the Numerical Rating Scale (NRS), and impact on quality of life through the Dermatology Life Quality Index (DLQI)). Thyroid dysfunction, including hypothyroidism and subclinical hypothyroidism, was assessed based on records from the Endocrinology Department. **Results**: Thyroid dysfunction was found in 4.2% of patients, all classified as hypothyroidism, primarily subclinical. The affected patients were generally older, with a mean age of 57.4 years. No significant differences in psoriasis severity (PASI, BSA) or treatment response were observed between patients with and without thyroid dysfunction. **Conclusion**: Our findings suggest hypothyroidism is the main thyroid dysfunction in psoriatic patients, independent of psoriasis severity. The lack of impact on psoriasis severity suggests hypothyroidism may be an independent comorbidity, warranting further research into shared inflammatory mechanisms.

## 1. Introduction

Psoriasis is a chronic inflammatory skin disorder driven by immune dysregulation, primarily mediated by T-helper (Th1 and Th17) cells, affecting around 2–3% of the global population. This disease involves both genetic predisposition and environmental factors, leading to an aberrant immune response that results in keratinocyte hyperproliferation and persistent skin inflammation. Beyond its dermatological manifestations, psoriasis is associated with an increased risk of systemic comorbidities, including metabolic, cardiovascular, and various autoimmune diseases. Among these, thyroid dysfunction has emerged as a notable comorbidity, though the underlying pathophysiological links between psoriasis and thyroid disorders remain incompletely understood [1].

Escribano-Serrano et al. [2] published, in 2016, the most up-to-date data to date on the prevalence of hypothyroidism in our region, Andalusia, Spain. Contextualizing existing epidemiological evidence is essential, particularly as it pertains to the population represented in our cohort. In their study, the authors aimed to estimate the prevalence of hypothyroidism by analyzing levothyroxine consumption during 2014 as a proxy indicator of disease burden. Due to the limited availability of epidemiological data on hypothyroidism in Spain, they utilized real-world prescription records from the Andalusian Public Health System to generate updated estimates and assess demographic and geographic variability. Data were obtained from pharmacy dispensing records, linked to unique patient identifiers for individuals who collected levothyroxine under public health coverage. The study population comprised over 8 million individuals, 96.8% of whom were registered with the public healthcare system. A total of 321,364 individuals were identified as levothyroxine users, corresponding to an overall hypothyroidism prevalence of 3.95%. The condition was significantly more prevalent in women (6.41%) compared to men (1.41%), with a female-to-male ratio of 4.5:1. Prevalence increased with age, particularly among women aged ≥60 years (11.37%). Geographic analysis revealed substantial variation across provinces and healthcare districts. Notably, approximately 27% of patients were classified as low-dose levothyroxine users (25–50 mcg/day), more commonly among older women, potentially reflecting treatment of subclinical hypothyroidism. While the study could not differentiate between clinical and subclinical forms, its primary strength lies in the use of large-scale, population-based prescription data, offering a valuable and cost-effective tool for epidemiological monitoring.

Autoimmune thyroid diseases (AITDs), such as Hashimoto’s thyroiditis and Graves’ disease, share immune-mediated pathways with psoriasis, particularly those involving cytokines like interleukin-17 (IL-17) and interleukin-23 (IL-23), which are prominent in both conditions. Additionally, these diseases display similar genetic and inflammatory markers, with shared pathways implicated in immune system dysregulation. For instance, anti-thyroid peroxidase antibodies (anti-TPO) and anti-thyroglobulin antibodies (anti-TG) are frequently observed in patients with AITDs and reflect a breakdown in immune tolerance, a mechanism also relevant in psoriasis pathogenesis [3].

While several studies have reported an increased incidence of thyroid dysfunction in individuals with psoriasis, the findings are inconsistent across populations and research designs. Some investigations highlight a higher prevalence of hypothyroidism and thyroid autoimmunity in psoriatic patients, suggesting a common inflammatory basis. In contrast, other studies fail to find a significant association, particularly when analyzing variables such as thyroid-stimulating hormone (TSH), free thyroxine (FT4), and thyroid autoantibodies among psoriatic and non-psoriatic populations. For example, recent longitudinal studies have demonstrated a trend toward elevated TSH levels in psoriatic patients, although without reaching statistical significance, leaving the association inconclusive [4,5].

The hypothesized link between thyroid hormones and psoriasis involves thyroid hormone receptors on keratinocytes, which influence epidermal growth factor and cellular proliferation in the skin. Elevated levels of triiodothyronine (T3) and thyroxine (T4) may exacerbate the hyperproliferative and inflammatory characteristics of psoriasis (1). Conversely, certain treatments for thyroid disorders have shown potential benefits in controlling psoriasis symptoms, suggesting an interaction between thyroid hormone homeostasis and psoriatic pathology. Antithyroid thioureylenes, such as propylthiouracil (PTU) and methimazole, are commonly used as first-line therapy for hyperthyroidism. Their potential in treating psoriasis has also been acknowledged for many years. A treatment course with PTU lasting six to eight weeks can significantly improve psoriatic lesions, as indicated by a reduction in the Psoriasis Area and Severity Index (PASI), through multiple mechanisms of action. Additionally, emerging evidence proposes that subclinical thyroid dysfunction could contribute to the progression or severity of psoriasis, further underscoring the relevance of assessing thyroid function in these patients [6].

By investigating this relationship in a cross-sectional sample of adults with moderate-to-severe psoriasis under treatment with biological therapy, we aim to clarify whether psoriasis predisposes individuals to thyroid disorders and to identify potential demographic or clinical factors influencing this association. The results could provide valuable insights into the pathophysiology shared between these two conditions and support a more integrative approach to managing patients with psoriasis, considering potential thyroid dysfunction in their clinical assessment.

## 2. Materials and Methods

This cross-sectional study included patients with moderate-to-severe psoriasis who were undergoing biologic therapy. Patients were recruited consecutively from the Psoriasis Unit of the Dermatology Department at Hospital Universitario San Cecilio in Granada, Spain, between 2017 and 2023. Only patients who provided informed consent participated in the study, and the Institutional Ethics Committee approved the protocol (Registration number: HUSC_DERM_2023_001).

### 2.1. Patient Selection

The inclusion criteria required patients to have a confirmed diagnosis of moderate-ate-to-severe psoriasis and to be receiving biological therapy.

To perform an effective comparison, data were collected from patients with mild psoriasis or those with moderate disease managed by conventional systemic treatments, such as methotrexate, cyclosporine, acitretin, or phototherapy, using the same clinical criteria. This segmentation allowed for a more specific focus on patients with higher disease severity who were candidates for advanced therapy.

In our hospital, all patients undergoing biological therapy for moderate-to-severe psoriasis must have previously received conventional systemic treatment (such as methotrexate, acitretin, or cyclosporine) or phototherapy. Therefore, these patients already meet the severity criteria equivalent to those who are prescribed systemic therapy in our setting. The aim was to characterize thyroid dysfunction prevalence specifically in patients with moderate-to-severe psoriasis on biologics.

### 2.2. Data Collection

Comprehensive demographic, clinical, and treatment-related data were collected for each participant. Demographic data included age, sex, body mass index (BMI), smoking status, and relevant comorbidities. Psoriasis severity was evaluated using the Psoriasis Area Severity Index (PASI), body surface area (BSA) involvement, Investigator’s Global Assessment (IGA), pruritus severity using the Numerical Rating Scale (NRS), and impact on quality of life through the Dermatology Life Quality Index (DLQI).

Clinical characteristics specific to thyroid function were assessed through the Endocrinology Department’s records, documenting diagnoses of thyroid dysfunction (hypothyroidism or subclinical hypothyroidism) including the time of onset of the disease. In hypothyroid patients, further classification was made into subtypes such as Hashimoto’s thyroiditis and subclinical hypothyroidism based on thyroid function tests and antibody profiles.

### 2.3. Thyroid Function Assessment

Routine thyroid function tests were conducted to assess thyroid health, measuring serum levels of thyroid-stimulating hormone (TSH) and free thyroxine (FT4), as well as thyroid-specific autoantibodies, including anti-thyroid peroxidase (anti-TPO) and anti-thyroglobulin (anti-TG) antibodies. All the patients in the sample had their TSH levels determined at least once during the course of the disease. Therefore, the figures for thyroid dysfunction and the prevalence extracted in our study, although limited, are consistent with a cohort of patients with moderate–severe psoriasis in a Spanish tertiary hospital.

Subclinical hypothyroidism was defined by elevated TSH with normal FT4, while overt hypothyroidism was diagnosed in cases of high TSH and low FT4. According to the protocols established in our hospital, none of the patients diagnosed with subclinical hypothyroidism were prescribed active treatment but they were subjected to closer monitoring.

Hashimoto’s thyroiditis was indicated by elevated anti-TPO antibodies. Only patients with confirmed moderate-to-severe psoriasis receiving biologic treatment were included in the thyroid dysfunction analysis.

The reference values used by our laboratory for the evaluated variables are as follows: thyroglobulin: 3.7–64.2 ng/mL; TSH: 0.34–5.1 mIU/mL; free T4 (fT4): 0.5–1.5 pg/mL; anti-thyroid peroxidase antibodies: 0–5.6 IU/mL; TSH receptor antibodies: 0–3.1 IU/L; and anti-thyroglobulin antibodies: 0–4.1 IU/mL.

### 2.4. Data Analysis

The primary goal was to assess the prevalence of thyroid dysfunction in patients with moderate-to-severe psoriasis under biologic therapy. Secondary objectives included comparing demographic and clinical characteristics between psoriatic patients with thyroid dysfunction and those without thyroid conditions.

Statistical analyses were performed using Python (Python Software Foundation; Beaverton, OR, USA, version 3.8) and the Pandas library (Austin, TX, USA). Descriptive statistics summarized the cohort’s demographic and clinical characteristics, with continuous variables presented as means ± standard deviations and categorical variables as counts and percentages. Comparative analyses between groups (with and without thyroid dysfunction) employed the t-test for continuous variables and the chi-square test for categorical variables.

To calculate the required sample size for estimating a proportion with a specified margin of error, we use the following formula:

$$n=\frac{Z^{2}\;p\;(1-p)}{E^{2}}$$

where:

- *Z* = 1.96 (for a 95% confidence level)
- *p* = 0.0424
- *E* = 0.05 (margin of error)

A minimum sample size of 63 patients was calculated as necessary to estimate the prevalence of hypothyroidism with a 95% confidence level and a margin of error of ±5%. Given that the study cohort included 401 patients, the sample size was more than sufficient to ensure adequate statistical power for prevalence estimation

### 2.5. Statistical Considerations

For all analyses, a *p*-value of <0.05 was set as the threshold for statistical significance.

## 3. Results

A total of 401 patients with moderate-to-severe psoriasis on biologic therapy were included in this study. Among them, 17 patients (4.2%) had a documented diagnosis of thyroid dysfunction, establishing a prevalence rate of 4.2% within this cohort. Of these, four cases had a prior diagnosis of thyroid dysfunction before being diagnosed with psoriasis.

### 3.1. Objective 1: Prevalence and Characteristics of Thyroid Dysfunction in Psoriatic Patients

In patients diagnosed with thyroid dysfunction, all cases were classified as hypothyroidism, specifically subclinical hypothyroidism (Table 1 and Table 2). Within this subgroup, demographic and clinical characteristics were as follows:

1. Type of Hypothyroidism: All 17 patients exhibited subclinical hypothyroidism, representing 100% of the cases within this group. One patient was diagnosed with multinodular goiter, three patients with Hashimoto’s thyroiditis, and the remaining patients with subclinical hypothyroidism.
2. Duration of Hypothyroidism: Among those with hypothyroidism, some cases had a documented history dating back to 2014 or earlier, suggesting a chronic progression of thyroid dysfunction over at least a decade in select patients.
3. Demographic Profile:
  - Mean Age: The mean age of patients with thyroid dysfunction was 57.4 years, with a range of 49.6 to 72.2 years.
  - Weight and Height: Patients in this group had a mean weight of 86.7 kg and an average height of 164.7 cm, suggesting that many individuals in this cohort fall within an overweight to mildly obese BMI range.
4. Additional Autoimmune Conditions: No other autoimmune diseases were present among patients with thyroid dysfunction, aside from their hypothyroidism.
5. Treatment and Follow-Up: All the patients in this subset adhered strictly to the prescribed psoriasis biologic therapy guidelines and did not use combined treatments. On average, these patients attended two follow-up visits per year for monitoring and management.

**Table 1:** Demographic characteristics of the hypothyroid subgroup.

Patient	Sex	Age	BMI	Smoking Habit	Comorbidities	Year Onset Psoriasis	Year Onset Hypothyroidism	Clinical Onset of Psoriasis
1	M	75	32	No	Diabetes mellitus, hypertension, dyslipidemia, metabolic syndrome, espondiloartrosis	1992	2001	Plaque
2	M	53	26	No	No	2019	2015	Plaque
3	F	44	39	No	Peripheral psoriasis arthritis, fibromyalgia	1993	2003	Plaque
4	F	49	52	No	Hypertension, peripheral psoriasis arthritis, anxiety–depressive syndrome	2003	2004	Plaque
5	F	50	22	No	Peripheral psoriasis arthritis, anxiety–depressive syndrome	1984	1999	Plaque
6	F	74	31	Yes	Dyslipidemia	2017	2006	Palmoplantar
7	F	50	30	No	Dyslipidemia	1990	1994	Plaque
8	F	53	30	Yes	Dyslipidemia, peripheral psoriasis arthritis, endometriosis	1984	1991	Palmoplantar
9	F	62	23	Yes	No	2011	2012	Plaque
10	F	74	41	Yes	Hypertension, dyslipidemia, metabolic syndrome, peripheral psoriasis arthritis, depression	2014	2001	Plaque
11	F	64	33	Yes	Hypertension, anxiety–depressive syndrome, multiple sclerosis	2015	2017	Pustulous palmoplantar
12	F	61	23	No	Hypertension	1968	1987	Plaque
13	F	72	42	No	Diabetes mellitus, hypertension, dyslipidemia, metabolic syndrome, peripheral psoriasis arthritis, anxiety–depressive syndrome	2015	2024	Plaque
14	F	66	27	No	No	2000	2002	Palmoplantar
15	F	79	32	No	Diabetes mellitus, hypertension, dyslipidemia, metabolic syndrome, heart failure	2016	2014	Scalp
16	F	41	26	No	No	1993	1996	Plaque
17	M	27	33	No	Inflammatory bowel disease	2009	2004	Scalp

M = Male; F = Female.

**Table 2:** Clinical characteristics of the hypothyroid subgroup at baseline.

Patient	PASI	BSA	DLQI	NRS Pruritus	Number of Therapeutic Lines Used (Systemic and Biological)	Current Treament	Time of Evolution with Treatment (Years)
1	8	10	11	5	Conventional Systemic 4, biological 2	Ixekizumab	4.3
2	6	8	5	0	Conventional Systemic 1, biological 1	Adalimumab bio	0.7
3	5	5	5	10	Conventional Systemic 2, biological 0	Apremilast	1.4
4	7	18	10	8	Conventional Systemic 2, biological 2	Secukinumab	4
5	10	10	12	9	Conventional Systemic 1, biological 1	Adalimumab bio	3
6	2	4	12	5	Conventional Systemic 3, biological 3	Secukinumab	4
7	12	14	8	2	Conventional Systemic 2, biological 2	Risankizumab	4
8	4	6	17	10	Conventional Systemic 1, biological 2	Secukinumab	1
9	7	9	10	6	Conventional Systemic 1, biological 1	Etanercept	2
10	17	23	17	7	Conventional Systemic 1, biological 1	Adalimumab bio	7
11	2	2	2	8	Conventional Systemic 2, biological 2	Guselkumab	1
12	6	7	10	0	Conventional Systemic 1, biological 2	Tildrakizumab	0
13	12	21	13	10	Conventional Systemic 3, biological 2	Secukinumab	4
14	3	4	23	10	Conventional Systemic 3, biological 0	Apremilast	0
15	9	14	22	10	Conventional Systemic 2, biological 1	Adalimumab bio	3
16	11	16	15	10	Conventional Systemic 1, biological 1	Adalimumab bio	3
17	5	9	12	8	Conventional Systemic 1, biological 3	Risankizumab	3

Psoriasis Area Severity Index (PASI), body surface area (BSA) involvement, Pruritus severity using the Numerical Rating Scale (NRS), Dermatology Life Quality Index (DLQI).

### 3.2. Objective 2: Comparison Between Patients with and Without Hypothyroidism

**Figure 1:**
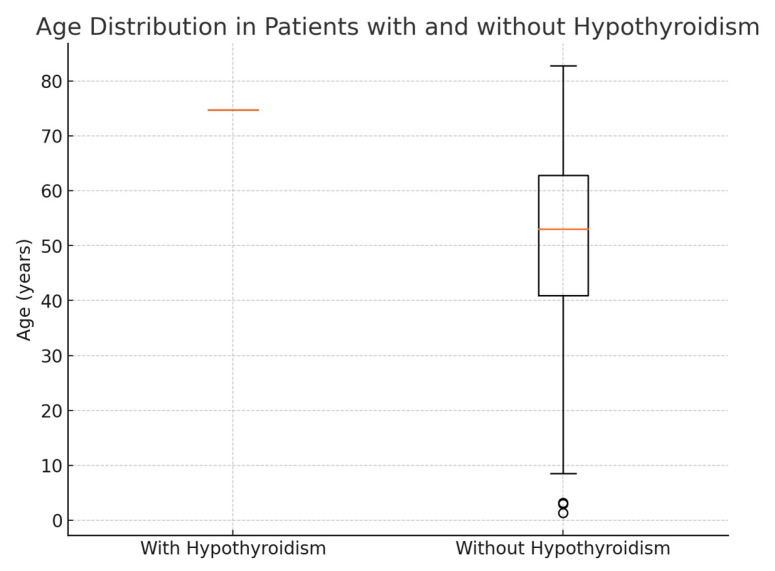
Age distribution in patients with and without hypothyroidism.

**Figure 2:**
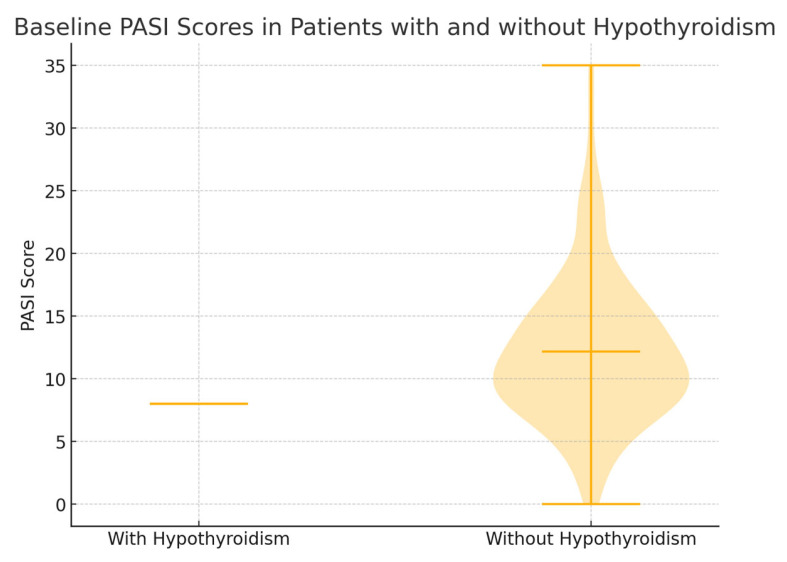
Baseline PASI scores in patients with and without hypothyroidism.

1. Demographic Characteristics
  - Age: Patients with hypothyroidism were generally older, with an average age of 74.8 years, compared to an average age of 51.5 years in patients without hypothyroidism. This age difference suggests that thyroid dysfunction in this population may be associated with older age (Figure 1).
  - The F:M ratio was 14:17, which meant that 82.35% of the patients with thyroid dysfunction in our study were female.
  - Weight and Height: Hypothyroid patients had a mean weight of 92 kg and an average height of 169 cm, whereas those without hypothyroidism averaged 82.8 kg and the same mean height of 169 cm.

2. Psoriasis Severity Parameters (Baseline and Over Time) (Figure 2)
  - Psoriasis Area Severity Index (PASI):
    ○ Baseline: Patients with hypothyroidism had a lower baseline PASI score (average of 8) compared to patients without hypothyroidism (average of 12.2), suggesting that the latter group had more severe psoriasis at the initial evaluation.
    ○ Five-Year Follow-Up: PASI scores showed a tendency to decrease in both groups over time, reflecting a positive response to biologic treatment across the cohort.
  - Body Surface Area (BSA):
    ○ Baseline: Patients with hypothyroidism had an average BSA involvement of 10%, while those without hypothyroidism averaged 17.3%, indicating more extensive body surface involvement in the non-hypothyroid group at baseline.
    ○ Evolution: Over the treatment period, BSA values generally decreased in both groups, demonstrating an improvement in skin involvement with consistent therapy.
  - Dermatology Life Quality Index (DLQI): Both groups experienced a reduction in DLQI scores over time, indicating enhanced quality of life with treatment. The initial DLQI values did not significantly differ between the groups, suggesting comparable quality of life impacts from psoriasis regardless of thyroid status.
  - Physician’s Global Assessment (PGA) and Pruritus Numerical Rating Scale (NRS): Both scores showed improvements in the severity and pruritus intensity in both groups, with similar initial and follow-up values.
  - No statistically significant correlation was observed between Psoriasis Area Severity Index (PASI) or Body Surface Area (BSA) involvement and serum levels of anti-thyroid peroxidase antibodies (anti-TPO) among patients with thyroid dysfunction (Pearson’s r < 0.1, *p* > 0.05). Likewise, no association was found between the dosage of thyroid hormone replacement therapy (levothyroxine) and psoriasis severity measures (PASI or BSA) in treated patients. These findings suggest that neither autoimmune thyroid antibody levels nor the intensity of hormonal correction correlate with cutaneous disease burden in this cohort.

3. Treatment Lines

The analysis showed that the group of patients without thyroid dysfunction had previously received an average of two conventional systemic treatments and 1.50 biologics. On the other hand, the group with thyroid dysfunction had received 1.82 conventional systemic treatments and 1.52 biologics, with no statistically significant differences between the two groups, indicating a comparable therapeutic history in terms of treatment switches or modifications over time. This consistency in the number of treatment lines suggests that patients, regardless of thyroid status, required similar levels of therapeutic intervention for psoriasis management.

### 3.3. Objective 3: Prevalence and Characteristics of Thyroid Dysfunction in Psoriatic Patients Receiving Conventional Systemic Therapy

The clinical and epidemiological characteristics of patients receiving conventional systemic therapy with a diagnosis of moderate psoriasis at our hospital during the time period evaluated are shown in Table 3.

## 4. Key Differences and Clinical Implications

The main differences observed between the groups with and without hypothyroidism were age and initial severity scores for psoriasis, with the hypothyroid group being older and presenting with less severe baseline psoriasis (as measured by PASI and BSA). Despite these baseline differences, the treatment outcomes, as reflected by improvements in PASI, BSA, DLQI, PGA, and pruritus scores, were similar between the groups, suggesting effective management of psoriasis with biologic therapy in both populations.

No statistically significant differences were observed between the groups regarding psoriasis severity parameters or the number of treatment lines. This lack of significant disparity implies that the presence of thyroid dysfunction, specifically hypothyroidism, does not substantially impact the severity or treatment responsiveness of psoriasis.

## 5. Discussion

This cross-sectional analysis examined two psoriasis populations drawn from the same tertiary center—734 adults with mainly moderate psoriasis treated with conventional systemic agents and 401 patients escalated to biologic therapy for moderate-to-severe disease—to explore whether disease severity or treatment modality affects thyroid status. The biologic cohort was almost a decade older on average (51.5 years vs. 42.6 years) and presented with approximately double the cutaneous burden (baseline PASI 12.2 vs. 5.4; BSA 17.3% vs. 7.6%), reflecting the higher inflammatory load that drives biologic use. In parallel, cumulative hypothyroidism prevalence was modestly higher among biologic-treated patients (4.2%) than in those receiving conventional agents (2.18% at treatment initiation plus 1.25% incident cases during follow-up, i.e., 3.43% overall). Strikingly, virtually all detected cases in both groups corresponded to subclinical hypothyroidism, and its presence did not appear to alter psoriasis severity trajectories, suggesting that thyroid dysfunction behaves as an independent comorbidity rather than a driver of cutaneous disease activity in this setting.

Our study’s prevalence of thyroid dysfunction among psoriasis patients aligns with a growing body of research suggesting an elevated incidence of thyroid disorders, particularly hypothyroidism, in individuals with psoriasis. A study by Yumnam et al. [7] reported deranged thyroid function in 19.8% of psoriatic patients, with hypothyroidism in 7.2% and subclinical hypothyroidism in 6.3%, indicating a significant occurrence of thyroid dysfunction in psoriatic populations compared to controls. Although these numbers differ from our observed prevalence, the consistency in identifying hypothyroidism as the predominant type corroborates our findings. Our data also indicate a slightly higher prevalence than the last study published in our area [2], but although an appropriate statistical inference cannot be established, it does indicate that it is a comorbidity to be considered and followed longitudinally in our patients.

On the other hand, some large cohort studies have not identified a strong association between psoriasis and thyroid dysfunction. For instance, Meneghini et al. [6], in the ELSA-Brasil study, observed no significant increase in thyroid dysfunction among psoriasis patients compared to non-psoriatic individuals. Moreover, they found a trend toward elevated TSH in psoriatic patients, particularly in women, yet this did not reach statistical significance. Such findings highlight the variability in reported associations, possibly due to differences in population genetics, environmental factors, and psoriasis severity.

Our study observed a low prevalence of thyroid autoimmunity markers, such as anti-thyroid peroxidase (TPO) antibodies, among the participants, which contrasts with the findings in other studies. For example, Rana et al. [8]. documented elevated anti-TPO antibodies in 13.5% of psoriatic patients, and Alidrisi et al. [9]. observed a significantly higher presence of anti-TPO and anti-thyroglobulin antibodies in psoriasis patients with Hashimoto’s thyroiditis. Although the low levels in our study may be attributable to the smaller cohort size, it raises questions regarding the mechanisms underlying thyroid autoimmunity in psoriatic populations. The shared immune pathways, particularly Th1- and Th17-mediated inflammation involving cytokines such as IL-17 and IL-23, are suspected to contribute to both psoriasis and thyroid autoimmunity.

Recent evidence indicates a clear age-related rise in subclinical hypothyroidism prevalence among elderly cohorts. A comprehensive narrative review (2024) highlights that subclinical hypothyroidism is “frequently found in older individuals,” noting divergent guideline recommendations and emphasizing that incidence increases notably in groups aged ≥65 years [10]. A longitudinal analysis pooling community-dwelling participants aged 65 and older across multiple European countries found that TSH levels normalized in only 39.9% after one year, implying a substantial and persistent prevalence of subclinical hypothyroidism in this age group [11]. In our cohort, age emerged as a significant demographic factor in psoriatic patients with thyroid dysfunction, with older patients more likely to exhibit hypothyroidism. This finding resonates with the literature highlighting age as a critical risk factor for thyroid dysfunction in psoriasis. Furthermore, our study did not find significant differences in psoriasis severity, as measured by PASI and BSA, between patients with and without thyroid dysfunction. This aligns with studies by Vassilatou et al. [12] and others, who reported no significant correlation between thyroid dysfunction and psoriasis severity. Notably, our hypothyroid patients had a lower baseline PASI score, suggesting that thyroid dysfunction may not exacerbate psoriasis severity directly but could represent a concurrent comorbidity more prevalent in older or otherwise predisposed individuals.

The immune-mediated inflammation seen in both psoriasis and thyroid dysfunction could explain the observed comorbidity, with specific cytokines and cellular pathways playing a crucial role. IL-23 and IL-17 are central to the inflammatory process in psoriasis and are similarly implicated in autoimmune thyroid diseases (AITD), such as Hashimoto’s thyroiditis [13]. IL-17 plays a pivotal role by promoting keratinocyte-derived chemokines, leading to chronic skin inflammation and hyperproliferation. IL-17 is also implicated in autoimmune thyroiditis. Additional chemokines, such as CXCL10, CXCL9, CCL2, and CCL22, are expressed in both psoriatic and thyroid autoimmune diseases. The shift from Th1 to Th2 immune response, marked by CCL2 and CCL22, is observed in advanced stages of PsA and Graves’ disease. Moreover, NF-κB pathway dysregulation—a key feature in autoimmunity—occurs in both AITD and psoriasis. Psoriatic lesions also demonstrate disrupted thyroid and retinoid signaling [14]. Beyond the statistical association observed, the mechanistic underpinnings linking psoriasis and thyroid dysfunction likely stem from shared immunological pathways, particularly those involving Th17 cells, IL-17, and IL-23 cytokines, which contribute to both psoriatic inflammation and autoimmune thyroiditis. The overexpression of chemokines such as CXCL10 and dysregulation of the NF-κB signaling pathway further suggest a convergent inflammatory profile. Although our study primarily included patients with moderate-to-severe psoriasis on biologic therapy, careful attention was paid to minimize confounding by excluding patients with coexisting autoimmune conditions and rigorously collecting data on metabolic comorbidities, including BMI, hypertension, diabetes, and dyslipidemia. These variables were evenly distributed between the groups, allowing for a clearer interpretation of thyroid dysfunction as an independent comorbidity. Clinically, our findings underscore the importance of integrating routine thyroid function screening into the management of moderate-to-severe psoriasis, particularly in older patients or those with metabolic syndrome. Establishing standardized protocols, including baseline and periodic assessment of TSH, FT4, and anti-thyroid antibodies, may facilitate earlier detection and more holistic care in this complex patient population.

Oxidative stress, mediated by reactive oxygen species (ROS), is another significant contributor to psoriasis pathology, affecting cell proliferation, differentiation, and apoptosis. ROS accumulation is similarly seen in thyroid dysfunction, particularly hyperthyroidism. A study conducted at Qassim University (2014–2015) revealed that ROS-induced structural changes in human serum albumin (HSA) and thyroid antigens generate neo-epitopes, provoking autoimmune responses in psoriasis. The cross-reactivity of psoriasis-specific IgGs with ROS-modified thyroid antigens supports the hypothesis that oxidative stress contributes to thyroid-related autoimmunity in psoriatic patients [15].

Moreover, the involvement of thyroid hormone receptors in keratinocyte proliferation and epidermal growth factor signaling suggests that thyroid hormones might influence psoriasis pathogenesis, potentially exacerbating hyperproliferative aspects in psoriatic lesions [16,17].

## 6. Conclusions

Our findings add to the understanding of thyroid dysfunction (subclinical hypothyroidism) as a potential comorbidity in moderate-to-severe psoriasis. Although no significant impact on psoriasis severity was observed, the elevated risk of hypothyroidism in older patients with psoriasis warrants further exploration, particularly concerning the shared inflammatory pathways and potential genetic predispositions. Managing psoriatic patients may benefit from routine thyroid function screening, especially in older patients or those with clinical signs of thyroid dysfunction. Further studies should investigate whether targeted treatments addressing shared inflammatory pathways could mitigate both psoriatic and thyroid symptoms, offering a more holistic approach to managing patients with these complex, interrelated conditions.

## 7. Limitations and Future Directions

The limitations of our study must be considered when interpreting the findings. As a single-center analysis limited to patients with moderate-to-severe psoriasis undergoing biologic therapy, the generalizability of our results to broader psoriatic populations may be constrained. Additionally, the relatively small number of patients with hypothyroidism reduces statistical power and may obscure subtler associations between psoriasis and thyroid dysfunction. An important methodological consideration is that all patients receiving biologics in our cohort were previously treated with conventional systemic therapies, in accordance with national treatment guidelines. This prerequisite introduces a potential selection bias and raises the possibility that the observed prevalence of hypothyroidism in the biologic group may be overestimated, as these patients had already undergone prior systemic exposure and likely represent a more clinically complex subgroup. Future studies should aim to include larger and more heterogeneous cohorts spanning a wider range of psoriasis severities and treatment pathways. Moreover, longitudinal designs are essential to determine the temporal sequence between psoriasis onset and thyroid dysfunction, which may help clarify whether thyroid abnormalities act as a contributing factor to disease progression or simply arise as comorbidities over time.

## Figures and Tables

**Table 3:** Prevalence and Characteristics of Thyroid Dysfunction in Psoriatic Patients Receiving Conventional Systemic Therapy.

Demographic Characteristics (*n* = 734)	Average/Percentage
Age	42.6 years
Gender (Male/Female)	56%/44%
BMI	29.36
Smoking status	36%
Comorbidities	
Hypertension	27%
Dyslipidemia	19%
Diabetes	12%
Psoriatic arthritis	29%
Initial treatment	
Methotrexate	81%
Cyclosporine	6%
Acitretin	4%
Phototherapy (NB-UVB)	9%
Time of evolution of psoriasis	19.6 years
**Clinical features**	
PASI	5.4
BSA	7.6
IGA	2.3
NRS pruritus	4.5
DLQI	7.8
**Clinical features of thyroid dysfunction**	
Prevalence of hypothyroidism at the time of initiation of systemic therapy	n = 16 (2.18%)
Development of hypothyroidism in the observation period after psoriatic treatment	N = 9 (1.25%)
Average age of onset of thyroid dysfunction	51.7 years

## Data Availability

The raw data supporting the conclusions of this article will be made available by the authors on request.
